# A Virtual Reality App Intervention to Improve Concussion Recognition and Reporting in Athletes Aged 9 to 12 Years: Development and Pilot Testing

**DOI:** 10.2196/43015

**Published:** 2023-05-26

**Authors:** Lindsay Sullivan, Lara B McKenzie, Kristin Roberts, Robyn Recker, David C Schwebel, Thomas Pommering, Jingzhen Yang

**Affiliations:** 1 School of Health and Rehabilitation Sciences College of Medicine The Ohio State University Columbus, OH United States; 2 Center for Injury Research and Policy Abigail Wexner Research Institute Nationwide Children's Hospital Columbus, OH United States; 3 Division of Epidemiology College of Public Health The Ohio State University Columbus, OH United States; 4 Department of Pediatrics College of Medicine The Ohio State University Columbus, OH United States; 5 Department of Psychology University of Alabama at Birmingham Birmingham, AL United States; 6 Division of Sports Medicine Nationwide Children's Hospital Columbus, OH United States

**Keywords:** concussion, education, sports, athlete, athletic, virtual reality, youth, child, pediatric, head injury, symptom reporting, symptom recognition, patient education, brain injury, user experience, user centered design

## Abstract

**Background:**

Existing concussion education programs for preteen athletes typically do not result in sustained improvements in concussion symptom recognition or reporting behaviors. Virtual reality (VR) technology offers an innovative tool that may improve concussion symptom recognition and reporting behaviors among preteen athletes.

**Objective:**

We aimed to describe the design and development of a VR concussion education app, Make Play Safe (MPS), and present findings on the usability and preliminary efficacy of MPS in improving concussion recognition and reporting intentions among soccer athletes aged 9-12 years.

**Methods:**

A collaborative user-centered design process was implemented to develop and evaluate MPS, a semi-immersive VR concussion education app designed to address two behavioral outcomes in preteen athletes aged 9-12 years: (1) recognizing concussion and (2) reporting concussion. The development of MPS occurred in three phases: (1) design and development, (2) usability testing, and (3) preliminary efficacy testing. During phase 1, consultations were completed with 6 experts. Additionally, 5 interviews with children who had a history of concussion were conducted to collect feedback about the proof of concept of MPS. During phase 2, a participatory workshop with 11 preteen athletes and a small group discussion with 6 parents and 2 coaches were conducted to explore the usefulness and acceptability of MPS from the perspective of end users. Finally, phase 3 included preliminary efficacy testing with 33 soccer athletes aged 9-12 years to examine changes in concussion-related knowledge, attitudes, and reporting intentions from pre- to postintervention. The data generated from each phase of this study informed the development of the final version of the proof of concept of the VR concussion education app, MPS.

**Results:**

Experts positively rated the features of MPS and noted that the design and content were innovative and age-appropriate. Preteens with a history of concussion indicated the scenarios and symptoms portrayed in the app represented well what they experienced while concussed. Further, they stated that the app would be an engaging way for children to learn about concussions. The 11 healthy children in the workshop perceived the app positively, noting that the scenarios were informative and engaging. Results from preliminary efficacy testing revealed increases in many athletes’ knowledge and reporting intentions from pre- to postintervention. Others demonstrated no significant changes or a decrease in knowledge, attitudes, or reporting intentions from pre- to postintervention. Group-level changes in concussion knowledge and intention to report concussions were statistically significant (*P*<.05), while changes in attitudes toward reporting concussions were not (*P*=.08).

**Conclusions:**

Results suggest VR technology may be an effective and efficient tool to equip preteen athletes with the requisite knowledge and skills to recognize and report future concussions. Further research is recommended to examine the use of VR as an effective strategy to improve concussion-reporting behaviors in preteen athletes.

## Introduction

Organized sports are extremely popular among children, with approximately 36 million US school-aged children participating in at least one organized sport each year [1]. Sports participation provides numerous health benefits to children [2], including increased physical activity levels [3], improved cardiovascular fitness [4-6], increased quality of life [7], and improved mental health and well-being [8,9]. However, there are some negative aspects of sports participation [10], including most prominently the risk of sport-related injuries such as concussion [11,12].

Concussion, a type of mild traumatic brain injury, is a public health concern among children and adolescent athletes [13]. Approximately 2 million US children aged ≤18 years are estimated to experience sports- or recreation-related concussions each year [14]. This is likely an underestimate of the true incidence due to the large number of concussions that go unreported and therefore undiagnosed and uncounted, an observation supported by studies suggesting that up to 50% of sports- or recreation-related concussions in US children are unreported each year [15-17]. Failure to recognize and report a concussion may place athletes at increased risk for further injury that leads to short- and long-term consequences involving impaired thinking, language, learning, behavior, or emotions [18,19].

Multiple strategies, including both primary and secondary prevention strategies, have been designed and implemented to prevent concussions in sports and change the culture of concussion reporting [20,21]. One strategy that is widely adopted in the United States to promote concussion safety is concussion education. In fact, concussion education for children and adolescent athletes is a key tenet of current US state-level concussion laws, which are mandated by all 50 states [22,23]. Concussion education programs generally aim to increase knowledge and awareness of the signs and symptoms of concussion and promote concussion-reporting behaviors [24-26]. Most existing concussion education programs are delivered through traditional approaches such as informational handouts or educational sessions delivered in person or digitally [24-26].

While these traditional approaches can help children and adolescents learn about concussions [24-26], they typically do not result in sustained improvements in concussion-related knowledge, attitudes, or reporting intentions and are considered by experts to be largely ineffective in changing concussion-reporting behaviors [24]. Moreover, despite the widespread adoption of concussion education programs in the United States and internationally, existing programs have several shortcomings. First, most programs target children and adolescents aged 12-18 years old rather than younger children, despite younger children’s high level of participation in sports and documented risk for concussive injury. Second, there is a scarcity of theory-driven programs; evidence suggests that theory-driven programs are more effective than those that lack a theoretical underpinning [27]. Third, concussion education programs that use active and experiential learning strategies to teach preteens about concussions and the importance of concussion reporting are rare. These limitations highlight the need for different approaches to concussion education for preteen athletes. To enhance learning, such approaches should be theory-driven, engaging, innovative, and captivating for athletes of all ages.

Virtual reality (VR) technology, which enables users to have a multisensory experience in a simulated and all-encompassing interactive 3D world, offers several advantages over traditional and existing approaches to concussion education. First, it engages preteens by allowing them to “experience” concussive events and symptoms without the risk of actual injury in a safe, computer-generated, immersive, and 3D environment that resembles the real world. Second, VR technology is underpinned by the principles of the Experiential Learning Model, which emphasizes the importance of learning by doing and posits that knowledge is constructed through physical interaction [28,29]. It also capitalizes on the principles of future-oriented thinking, which suggest that children can develop the ability to think about the future and anticipate future states and needs to successfully plan and function in society [30,31]. Third, VR technology can be tailored to the individual participant, a feature that boosts both engagement and comprehension. Fourth, VR provides a fun and engaging learning environment.

VR technology has been extensively used in other health domains, including child pedestrian safety training [32-35], the neuropsychological rehabilitation of pediatric traumatic brain injury patients [36,37], and balance training for children with cerebral palsy [38,39]. However, no published study reports on using VR technology to educate preteens about concussions. This paper describes the design and development of a VR concussion education app intervention, Make Play Safe (MPS), and presents findings on the usability and preliminary efficacy of MPS in improving concussion symptom recognition and reporting intentions among soccer athletes aged 9-12 years old.

## Methods

### The MPS VR App

MPS is a semi-immersive VR concussion education app designed to address two primary behavioral outcomes of athletes aged 9-12 years: (1) recognizing concussion signs and symptoms and (2) reporting a suspected or actual concussion to an appropriate adult (eg, parent or coach). The version of the MPS app developed and then evaluated in this report begins with a brief introduction to concussion, providing the definition of concussion, describing concussion signs and symptoms, and explaining why it is important to report concussion symptoms to an appropriate adult. After the introduction, app users are directed through a scenario in which they virtually experience a concussive event while playing soccer. Specifically, preteens are immersed in the virtual environment as a soccer player playing on an outdoor soccer field when a virtual soccer ball unexpectedly hits them in the head, causing them to start experiencing various simulated concussion symptoms (eg, blurred vision, light sensitivity, headache, and nausea) both on the field and after virtually returning home. Filmed video clips and scenarios of actual preteen soccer players reinforce the virtual-to-physical relationship by allowing users to see familiar, relatable projections of themselves and their environment in the virtual space.

### Overview of the Design and Development of MPS

**Figure 1:**
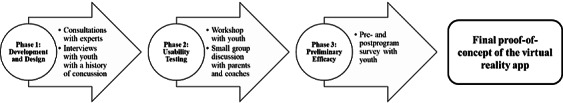
Design and development of Make Play Safe: a virtual reality app.

MPS was developed using a collaborative user-centered design process that involved VR experts, a clinician-researcher with expertise in concussion assessment and management, injury prevention scientists, psychologists with expertise in developmental and educational psychology, and a health education specialist. As detailed below, development occurred in three phases: (1) design and development, (2) usability testing, and (3) preliminary efficacy testing (Figure 1).

Before the development of MPS, a literature review on existing concussion education programs was conducted. Shortcomings and lessons learned were noted to guide the development of the initial proof of concept. The MPS app was designed for athletes aged 9-12 years, with an initial focus on soccer athletes, as soccer is a sport with a high risk of concussion [40] and a sport played by both girls and boys worldwide. MPS was developed for delivery via a mobile app and an accompanying VR headset.

### Phase I: Design and Development of MPS

Phase 1 identified the key features of effective VR technology needed to ensure the MPS app simulated lifelike concussion symptoms and scenarios.

#### Consultations With Experts

Six consultations with experts in design, communication, developmental psychology, sports medicine, and technology were conducted. These interviews had two goals: (1) to collect feedback about the initial proof of concept MPS app, including the appropriateness of the information for the target age group and relevance to pediatric concussion, and (2) to elicit recommendations on how to improve the visual and sensory design, the content, and the scenarios presented in the proof of concept app. The consultations took place from December 2019 to January 2020. Experts were identified by the research team.

Prior to the consultation, experts completed a preconsultation questionnaire that solicited recommendations for essential elements and features of VR apps as well as how to make VR apps available to and engaging for the target audience. During the consultations, experts viewed the proof of concept MPS app and then provided feedback on the app, including its features, the simulation of concussion symptoms, and the scenarios presented. Finally, after viewing the proof of concept of MPS, experts completed an app experience form to rate their experiences with MPS on a 5-point scale (1=positive to 5=negative), to describe their emotions while using the app, and to indicate the features they “liked most” and “liked least.” All consultations were conducted by a qualitatively trained member of our research team and were audio-recorded and transcribed.

#### Interviews With Children and Adolescents With a History of Concussion

Five interviews with children and adolescents (aged 11-18 years; 3 boys and 2 girls) who had a history of a physician-diagnosed concussion were conducted. The purpose of these interviews was threefold: (1) to collect feedback about the proof of concept MPS app (eg, elements they enjoyed), (2) to examine the accuracy of the concussion symptoms and scenarios presented in the proof of concept of MPS (eg, how much the presented symptoms and scenarios reflected participants’ actual concussion experiences), and (3) elicit recommendations on how the visual design, content, and scenarios could be improved.

Participants were identified through other hospital concussion studies and invited to participate. Inclusion criteria included a diagnosis of concussion within the past 12 months and full recovery from their concussion by the time of their interview. Prior to each interview, written and verbal assent or consent was obtained from participating children and a parent or legal guardian, respectively. Children and adolescents then viewed the MPS VR app session and provided feedback on MPS in four domains: (1) lessons about concussion, (2) their experience during the app, (3) the simulation of concussion symptoms, and (4) the design of the app. All interviews were conducted between November 2019 and January 2020 using an interview guide developed specifically for this purpose. Each interview lasted approximately 30 minutes and was audio-recorded and transcribed.

Based on feedback received during this phase of development, a prioritized list of app updates and feature design modifications was created. The highest-ranked priorities for modification included (1) adding onboarding instructions with a tutorial to familiarize users with the virtual environment and how to get started with the app, (2) enhancing the delivery of the symptoms directly simulated by the MPS app (eg, intensifying the simulated loud noises, bright lights, and blurriness) so that users can feel and experience them more, (3) adding age-appropriate educational interludes and a branching decision tree that leads to different outcomes depending on the decisions made within MPS (eg, if they choose to disclose concussion symptoms versus if they do not disclose symptoms), and (4) creating a conclusion or ending for the app that reiterates signs and symptoms of concussion and what to do if the user experiences a suspected concussion in the future.

### Phase II: Usability Testing

To evaluate the usability and acceptability of MPS by preteen athletes and their parents and coaches, a workshop with athletes and a small group discussion with parents and soccer coaches were conducted.

#### Workshop With Preteen Athletes

Soccer athletes aged 9-12 years old participated in an activity-based workshop designed to elicit design ideas and preferences for a VR app that educated preteens aged 9 to 12 years about sport-related concussions in a relevant and engaging way. A total of 11 preteen soccer athletes participated. Following written informed consent (parents) and assent (children), participants were assigned into small groups of 3-4 participants and guided through a series of activities. These activities included “Scenarios and Storytelling,” where children engaged in role-play portraying a concussed athlete and walked through the decision to disclose or not disclose symptoms of a suspected concussion; and “Virtual Reality Watching,” where each athlete individually viewed the proof of concept MPS app. After completing these activities, in researcher-led small group discussions, the preteens shared their views toward and acceptance of MPS as well as provided recommendations on how to improve the app. The 1-hour workshop was conducted at a local community center.

#### Small Group Discussion With Parents and Coaches

Six parents and 2 soccer coaches participated in a small group discussion led by members of our research team. Consent for participation and permission to take notes were obtained from each participant prior to the 40-minute small group discussion. After viewing the MPS proof of concept, parents and coaches took part in a script-guided discussion and were provided an opportunity to give general feedback on MPS. Discussion questions centered on parents’ and coaches’ views and experiences regarding preteen concussion education, perceived disadvantages for their child or team to receive concussion education via a VR app, and feedback on the current proof of concept of MPS, including visual app navigations, features, and core functionalities, as well as suggestions for app improvement.

### Phase III: Preliminary Efficacy

To evaluate the preliminary efficacy of MPS in improving concussion symptom recognition and reporting, a pre- or posttest design was conducted with 33 preteen athletes.

### Ethics Approval

All aspects of the study were approved by the institutional review board at Nationwide Children’s Hospital (IRB17-00260). Written informed consent (parents) and assent (children) were obtained from study participants prior to the study procedures. All participant data were deidentified to ensure the privacy and anonymity of participants. Participants were compensated with a US $25 ClinCard (Greenphire Inc) upon completion of the study.

### Procedure

Soccer leagues and clubs in central Ohio were contacted via email and asked to disseminate information about the study to families with a child aged 9-12 years participating in a soccer league. Participants were also recruited by word of mouth and snowball sampling. Interested families contacted the research team to learn more about the study. Following eligibility screening, written consent and assent were obtained from interested parents or guardians and preteens, respectively. Children aged 9-12 years were eligible if they played organized soccer in the past 12 months and spoke English.

A 1-group pre- and posttest design was used with 33 children. Children completed a preintervention survey to examine concussion-related knowledge, attitudes toward concussion reporting, and reporting intentions. They then completed the 10-minute MPS VR educational session using a VR headset and app, with guidance provided by a researcher as needed. After completing the training, children completed the postintervention survey assessing concussion-related knowledge, attitudes toward concussion reporting, and reporting intentions. Data were collected from June to December 2021.

### Measures

Concussion knowledge was assessed pre- and postintervention using an adapted version of the Rosenbaum Concussion Knowledge and Attitudes Survey–Students (RoCKAS-ST) [41]. The survey was pilot-tested prior to use in this study. Children were presented with 17 true-false questions about the detection, assessment, and management of sports-related concussions (eg, “A concussion can only happen if there is a direct hit to the head”). A total concussion knowledge score was calculated by summing the total number of correct responses to the true-false questions, with a higher score indicating greater concussion-related knowledge (possible range 0-17).

Attitudes toward concussion reporting were assessed pre- and postintervention. Participants rated their views toward 8 statements (eg, “It is important to tell my coach and parents when I think I have a concussion” and “It is necessary to tell my coach and parents when I think I have a concussion”) using a 5-point scale from 1=strongly disagree to 5=strongly agree. A total attitude score was calculated by summing the 8 responses (possible range 8-40), with higher scores indicating more favorable attitudes toward concussion reporting. These questions were developed by pediatric concussion researchers and pilot-tested prior to use in this study.

Concussion reporting intentions were measured using the reporting behaviors and intentions section of the RoCKAS-ST [41]. Children were presented with a written description of 3 scenarios and rated the following five items for each scenario on a 5-point scale from 1=strongly disagree to 5=strongly agree: (1) intention to report concussion to an adult (eg, coach or parent), (2) level of comfort with concussion reporting, (3) perceived support from their coach or parent to report a concussion, (4) willingness to be removed from play following a suspected concussion, and (5) playing the game as planned after a concussion. Participants were asked to answer each question as if they were the child in the scenario presented to them. A total concussion reporting intentions score was calculated by summing the participants’ responses to the 3 scenarios (possible range 15-75), with higher scores indicating greater concussion reporting intentions.

The following demographic variables were also collected from participants: sex, age, medical history of concussion (yes or no), and prior concussion education received (yes or no).

### Statistical Analyses

Demographic characteristics of the preteens were described, and descriptive data were considered. The methods of open coding [42], axial coding [43], and selective coding [44] were used to analyze the qualitative data generated during phases 1 and 2 of this study. Two trained coders independently reviewed and coded the transcripts to identify common themes and patterns across the consultations with experts, interviews with children and adolescents with a history of concussion, and small group discussions with parents and coaches, respectively. The themes identified focused on the strengths of the prototype and areas for improvement, as well as views toward the usefulness and accessibility of using VR technology to teach athletes about concussions. To examine the preliminary efficacy of MPS, mean scores, as well as percent score changes in outcomes of interest (ie, concussion knowledge, attitudes toward concussion reporting, and reporting intentions), were described. Paired tests were used to assess changes in scores from pre- to posttests. All quantitative analyses were conducted using SAS (version 9.4; SAS Institute Inc), and the significance level was set at α=.05.

## Results

### Phase I: Design and Development

Table 1 summarizes feedback from the experts and children and adolescents with a history of concussion, respectively; this feedback was used to update the initial proof of concept for the next phases of the project. Recommendations from experts included making the content visually appealing and easy to use, including incorporating interactive components to support the educational lessons, tailoring the content to the app user, and using gamification. Most experts reported that they had a positive experience with the proof of concept app and that they were entertained, informed, and engaged by the app. The most liked aspects of the MPS app included the general design, the realistic scenarios and visuals presented, and the quality of the audio and video footage. The least liked aspects included the headset used to deliver the VR experience, the app’s icons, and the fuzziness of some simulated symptoms (Table 1). The experts rated the visual and sensory features of MPS positively and felt the design and content of the MPS app were innovative and appropriate for the target age group of 9-12 years.

Children and adolescents with a history of concussion indicated that the scenarios and symptoms portrayed in MPS were very similar to those they experienced in real life. They reported the app would be a helpful and engaging way for preteen athletes to learn about concussions because it allowed them to experience various real-world simulated concussion symptoms both on the simulated soccer field (immediately after a virtual injury) as well as after returning to their virtual home. Participants also provided some recommendations on how the app could be improved, such as by adding instructions on how to navigate the app (Table 1).

**Table 1:** Feedback on the initial proof of concept of Make Play Safe (MPS).

Elements of the app		Feedback	
		Experts (n=6)	Children and adolescents with a history of concussion (n=5)
**General feedback**			
	Strengths	Presented age-appropriate and developmentally appropriate information Depicted life-like situations following concussion	Taught children about concussion and how to appropriately respond to concussion
	Areas for improvement	Give users the opportunity to see, hear, and experience concussion symptoms Provide guidance on what to do if a concussion is suspected	Provide instructions on how to navigate the app Increase the length of the scenarios
**Portrayal of signs and symptoms**			
	Strengths	Portrayed concussion symptoms accurately	Depicted symptoms and scenarios that were realistic and like those experienced in real life
	Areas for improvement	Provide more active engagement for viewers Intensify the symptoms	Simulate additional symptoms such as noise sensitivity, light sensitivity, balance problems and memory issues
**Design of the app**			
	Strengths	Used high quality audio and video	Gave options within the scenarios presented
	Areas for improvement	Collect information about the user Evaluate the users’ knowledge about concussion before and after using the app Provide instructions on how to engage with the app	Include a scenario depicting what happens in the first few days post injury as well as displaying potential consequences of not disclosing concussion symptoms Emphasize need to report concussion to an appropriate adult

### Phase II: Usability Testing

Preteen athletes in the workshop were receptive to a VR app that taught them concussion signs and symptoms and the importance of concussion reporting. They indicated that they liked the MPS VR session because the scenarios presented were realistic, making them feel like they were on an actual soccer field. They also reported that they enjoyed being able to experience concussion signs and symptoms in a virtual environment. Data collected from end users were incorporated into the design of the updated proof of concept, as described below.

Parents and coaches in the small group discussion reported that they enjoyed the storyline portrayed in the MPS app and felt that the MPS educational session was informative, realistic, and age-appropriate. Parents and coaches reported that the MPS app would prepare children to successfully recognize and respond to future concussions. Parents and coaches also noted that the completion of MPS would help facilitate communication about concussion safety, which may enhance children’s understanding of the importance of concussion reporting.

### Phase III: Preliminary Efficacy

A total of 33 preteen soccer players participated, including 20 (61%) boys and 13 (39%) girls. Of the 33 participants, 14 (42%) were 9 years old, 10 (30%) were 10 years old, 5 (15%) were 11 years old, and 4 (12%) were 12 years old. The mean concussion knowledge score significantly increased from 12.2 (SD 1.9) to 12.9 (SD 1.9) from pre- to postintervention. An increase in concussion knowledge from pre- to postintervention was demonstrated by 18 (55%) participants, whereas 7 (21%) demonstrated no change in concussion knowledge and 8 (24%) showed a decrease in concussion knowledge during the same period (Table 2).

There was no statistically significant increase in mean attitudes toward concussion reporting scores from pre- (mean 21.0, SD 2.8) to postintervention (mean 21.9, SD 3.1), *P*=.08. More favorable attitudes toward concussion reporting from pre- to postintervention were demonstrated by 17 (52%) preteens, 7 (21%) showed no change in attitudes, and 9 (27%) reported less favorable attitudes toward concussion reporting following the intervention.

Finally, there was a significant increase in mean concussion reporting intention scores from pre- (mean 56.7, SD 5.4) to postintervention (mean 58.7, SD 5.0). Increased intentions to report future concussions from pre- to postintervention were reported by 16 (49%) participants, while 7 (21%) showed no change in reporting intentions, and 10 (30%) reported decreased concussion reporting intentions following the intervention.

**Table 2:** Changes in symptom recognition, attitudes toward reporting, and intention to report from pre- to posttest (N=33).

Outcome	Decreased score, n (%)	No score change, n (%)	Increased score, n (%)	Pretest score, mean (SD)	Posttest score, mean (SD)	*P* value^a^
Concussion knowledge	8 (24)	7 (21)	18 (55)	12.2 (1.9)	12.9 (1.9)	.04
Attitudes toward concussion reporting	9 (27)	7 (21)	17 (52)	21.0 (2.8)	21.9 (3.1)	.08
Reporting intentions	10 (30)	7 (21)	16 (49)	56.7 (5.4)	58.7 (5.0)	.02

^a^*P* value was based on paired test.

## Discussion

### Key Findings

This pilot study reports the 3-phase process to design, develop, and examine the feasibility and preliminary efficacy of MPS, a VR concussion education app. We used a systematic and iterative process to develop a proof of concept for the VR app intervention. Our findings showed that it was well accepted by preteen athletes as well as their parents and coaches. The findings also showed that the intervention had a positive effect on many preteen athletes’ concussion-related knowledge and reporting intentions immediately following the concussion education VR intervention. These results provide support for the continued development, refinement, and evaluation of MPS as a VR app to educate and train preteen athletes to recognize and report signs and symptoms of concussion.

The use of VR technology to teach preteens about concussions offers many potential advantages over traditional approaches to concussion education. First, unlike traditional concussion education approaches, VR technology allows athletes to virtually feel and experience what it is like to have a concussion in a safe, computer-generated environment [45,46]. VR allows interventionists to directly simulate concussion symptoms like dizziness and blurry vision and to indirectly convey nonsensory symptoms like headache and vomiting through scenarios and stories. These manipulations provide an impactful, experiential, and active learning environment for children, allowing them to enhance their knowledge through experience. Prior studies demonstrate that experiential and active learning strategies increase engagement and add value to learning experiences [25-32]. Another advantage of VR technology over other approaches to concussion education is that it can be rapidly and widely disseminated. Unlike many other forms of concussion education, VR technology does not require personnel to deliver the concussion education session; children can experience the education session via a smartphone-based VR app at any time and place convenient to them. This provides opportunities for wide accessibility [47].

Our findings highlight the value of using a systematic and collaborative user-centered approach to design and develop intervention programs like VR technology for children. Active engagement from experts and end users contributed valuable insights that guided the iterative development of our proof of concept app, including recommendations on how to make the app more engaging and to increase the realism of the concussion symptoms simulated within the app. The final proof of concept app was accepted by our target audience and offered an engaging strategy to equip preteens with the requisite knowledge, skills, attitudes, and intentions to recognize and respond to future concussions. The participatory approach used to design and develop MPS could be applied to the development of other interventions for children, including those incorporating VR technology.

We found improvements in many preteens’ concussion-related knowledge, attitudes toward reporting, and reporting intentions following the VR session, although the effect of the intervention on changes in attitudes from pre- to postintervention was not statistically significant. Prior studies suggest that increased concussion-related knowledge, favorable attitudes toward concussion reporting, and reporting intentions are associated with a decreased likelihood of continuing to play in a game or practice while symptomatic from a concussion [48,49]. However, it is important to note that we did not observe improvements in all study participants, with some demonstrating stable or decreased concussion-related knowledge, attitudes, and reporting intentions following the VR session. There are several possible explanations for this. One possibility is that the use of a single VR session was insufficient to motivate behavior change in all children; a higher dosage of the VR session may be necessary. A second possibility is that multicomponent, multilevel interventions are needed to improve athletes’ concussion-related knowledge, attitudes, and reporting intentions. The VR exposure might best be paired with supporting intervention components, such as input from parents or coaches. One final explanation for the findings is that educational content specifically tailored to age, gender, or other characteristics is required for preteen athletes. We used the same VR exposure for children between the ages of 9 and 12 years, both boys and girls, of all races and ethnicities. Tailoring the VR to help viewers associate with the characters might yield stronger results. Future studies with larger, more diverse samples of athletes are recommended to better understand the efficacy of VR-based concussion education programs like MPS.

### Limitations

Several limitations of this pilot study warrant attention. First, the sample size was small; the results may not generalize to other populations of athletes. Second, we did not include a comparison or control group. Third, we did not examine the effectiveness of the MPS app in improving actual concussion-reporting behaviors or monitor changes in concussion-related knowledge, attitudes toward reporting, or reporting intentions over time following the intervention. Prior to the broad-based implementation of concussion education via VR technology, future studies should determine the effectiveness and dosage of intervention required to improve preteens’ concussion symptom recognition and reporting behaviors using a more rigorous evaluation design with a larger sample. Fourth, the adapted survey we used was not validated in preteens aged 9-12 years. Psychometric research should be conducted to develop and validate instruments to examine concussion-related knowledge, attitudes, reporting intentions, and behaviors in this population of athletes.

### Conclusions

This study provides insights into how we can use VR technology to teach preteens about concussions as well as highlights the importance of using an iterative, user-centered approach when designing VR technology for preteens. Our findings suggest that VR technology may be an effective tool to teach preteens about concussions and thereby reduce the negative and potentially long-lasting health consequences of concussions by increasing concussion symptom recognition and reporting among preteen athletes. However, further research with a larger, more diverse sample is needed to determine the effectiveness of VR technology in improving concussion-reporting behaviors and ensure that such technology has a positive effect on preteens. This study provides a critical first step in understanding how VR technology can be used to improve concussion symptom recognition and reporting behaviors among preteen athletes and should be extended with further research.

## Abbreviations

MPS: Make Play Safe
RoCKAS-ST: Rosenbaum Concussion Knowledge and Attitudes Survey–Students
VR: virtual reality

## Data Availability

The data sets generated during or analyzed during this study are available to qualified users from the corresponding author on reasonable request.
